# Alzheimer’s Disease Association with Metals and Metalloids Concentration in Blood and Urine

**DOI:** 10.3390/ijerph19127309

**Published:** 2022-06-14

**Authors:** Loreta Strumylaite, Rima Kregzdyte, Odeta Kucikiene, Dale Baranauskiene, Vaida Simakauskiene, Rima Naginiene, Gyte Damuleviciene, Vita Lesauskaite, Reda Zemaitiene

**Affiliations:** 1 Neuroscience Institute, Medical Academy, Lithuanian University of Health Sciences, LT-50161 Kaunas, Lithuania; rima.kregzdyte@lsmuni.lt (R.K.); dale.baranauskiene2@lsmuni.lt (D.B.); vaida.simakauskiene@lsmuni.lt (V.S.); rima.naginiene@lsmuni.lt (R.N.); 2 Department of Geriatrics, Medical Academy, Lithuanian University of Health Science, LT-44307 Kaunas, Lithuania; odeta.kucikiene@lsmuni.lt (O.K.); gyte.damuleviciene@lsmuni.lt (G.D.); vita.lesauskaite@lsmuni.lt (V.L.); 3 Department of Ophthalmology, Medical Academy, Lithuanian University of Health Sciences, LT-50161 Kaunas, Lithuania; reda.zemaitiene@lsmuni.lt

**Keywords:** arsenic, chromium, selenium, Alzheimer’s disease

## Abstract

As there is some evidence that the risk for Alzheimer’s disease (AD) is partially attributable to environmental exposure to some metals and metalloids, we examined an association between AD and arsenic, chromium, and selenium in 53 AD patients and 217 controls. Urinary arsenic, blood chromium, and selenium were determined by inductively coupled plasma mass spectrometry. Logistic regression models calculating odds ratios (ORs) and 95% confidence intervals (CI) were used to estimate AD association with arsenic, chromium, and selenium. In AD patients, urinary arsenic and blood chromium were significantly higher, while blood selenium was significantly lower compared to controls. Increased blood selenium was related to a significant decrease in the odds of AD after adjustment for risk factors. Blood selenium per 1 kg × 10^−9^/m^3^ × 10^−4^ increment was associated with 1.4 times lower risk of AD (OR = 0.71; 95% CI 0.58–0.87). A significant increase in the odds of AD associated with increased blood chromium was also seen in the adjusted model: the OR per 1 kg × 10^−9^/m^3^ × 10^−3^ chromium increment was 2.39 (95% CI 1.32–4.31). The association of urinary arsenic with the risk of AD was not significant. The data obtained provide evidence that selenium reduces the risk of Alzheimer’s disease, while chromium increases it.

## 1. Introduction

Dementia in the aging population is becoming a worldwide issue and causes a significant health and economic burden. In 2015, about 47 million people all over the world lived with dementia, and this number is expected to triple by 2050 [1]. Alzheimer’s disease (AD) is the main form of dementia in the elderly. In 2020, the number of people with AD in Lithuania was 43,235, with morbidity rate of 15.47 per 1000 population [2].

AD is a multicausal, progressive, neurodegenerative disease that, according to the age at of onset of the disease and genetic predisposition, can be classified as early-onset familial or late-onset sporadic form of AD. Age and inheritance are the main risk factors for AD [3], but most cases of AD are considered to have a multifactorial etiology because of interactions between genetic, lifestyle, and environmental factors [4] such as low education, female gender, diseases (hypertension, cardiovascular diseases, stroke, diabetes mellitus, depression, and brain trauma), lifestyle (lack of exercise, obesity, alcohol use, and smoking), and environmental factors (pesticides, organic solvents, metals/metalloids) [5,6,7].

The main pathological features of AD are senile plaques and neurofibrillary tangles associated with the accumulation of amyloid-β (Aβ) and hyperphosphorylation of Tau protein in the brains [8]. However, besides formation of Aβ and Tau, AD development involves different cellular processes, such as the endoplasmic reticulum stress [9,10], oxidative stress that underlies neurotoxicity related neuronal loss [11], neuroinflammation [12,13], impaired glucose metabolism related to insulin/insulin-like growth factors resistance [14,15], and glial function [16,17]. In AD pathogenesis, all these processes are related to each other directly and/or indirectly, for instance, Aβ can act either as an antioxidant or a pro-oxidant according to its redox properties [18].

Metals and metalloids play an important role in the development of AD. Iron, copper, and zinc promote Aβ aggregation and plaque formation as Aβ is a metalloprotein [19,20,21]. Beta-site amyloid precursor protein cleaving enzyme 1 is a target of environmental neurotoxicants [22]. In vivo and in vitro studies have shown that the Aβ protein aggregation or increased level of Tau protein hyperphosphorylation is related to long-term exposure to neurotoxic metals/metalloids such as cadmium, mercury, lead, and arsenic [23,24,25,26,27,28,29,30]. Disrupted homeostasis of redox active metals (iron, copper, chromium) is associated with oxidative stress and formation of reactive oxygen species inducing DNA damage, lipid peroxidation, protein modification, and other effects common to many diseases including AD. The redox inactive metals/metalloids such as cadmium, arsenic, and lead express their toxicity by binding to sulfhydryl groups and depletion of glutathione [31]. Meanwhile, selenium demonstrates neuroprotective, antioxidative effects [32], attenuates the intracellular toxicity of Tau protein and Aβ, and inhibits accumulation of advanced glycation end-products [15,33].

Essential metals such as iron, copper, zinc, manganese, and selenium, as well as non-essential metals such as lead, mercury, and aluminum, are the most studied in order to assess their relationship with AD. Meanwhile, studies on the AD association with arsenic and chromium are very limited and inconclusive [34]. Both arsenic and chromium are included in the list of substances to be investigated under the “Human Biological Monitoring Initiative” that develops human biological monitoring as a tool for health-related environmental monitoring and scientific policy support for safer chemicals management [35]. Thus, in this study, we aimed to assess the AD association with arsenic, chromium, and selenium concentration in blood and urine.

## 2. Materials and Methods

### 2.1. Study Design

A hospital-based case–control study of AD risk factors was performed between March 2018 and March 2020 in two Hospitals of Lithuanian University of Health Sciences. The cases (*n* = 53: men (*n* = 12) and women (*n* = 41)) were patients aged 68–94 years with AD diagnosed within the study period in the Department of Geriatrics. AD patients received neuropsychological examination and cognitive function assessment that included the mini-mental status examination and the clinical dementia rating scale (the Blessed Scale). AD diagnosis by a council of physicians that involved a geriatrician, neurologist, and psychiatrist was based on criteria defined and revised by the National Institute of Neurological and Communicative Disorders and Stroke, and Alzheimer’s Disease and Related Disorders Association [36,37]. Most patients (75.5%) were diagnosed with an atypical or a mixed form of AD. The controls (*n* = 217: men (*n* = 100) and women (*n* = 117)) were patients aged 53–93 years free from AD and dementia undergoing treatment for eye diseases such as cataract, glaucoma, optic neuritis, or keratitis in the Department of Ophthalmology. We received written informed consent from each individual before they participated in the study. The study was conducted in accordance with the Declaration of Helsinki. The study protocol was approved by the Kaunas Regional Biomedical Research Ethics Committee (12-03-2018, No. BE-2-6).

### 2.2. Questionnaire

Both AD patients and controls were asked by trained interviewers to answer questions on some demographic and socioeconomic characteristics, personal history of diseases, height and weight, family history of AD, and lifestyle factors (smoking, use of alcohol, physical activity). If AD patients did not respond to the questions, information was received from their caregivers and/or medical records. Information on personal history of diseases was compared with medical records, giving them priority.

### 2.3. Blood and Urine Sample Collection

We gave oral instructions on how to obtain blood and urine samples to nurses of the departments involved in a study, asking for approximately 20 mL of blood to special vacutainers and 10–30 m^3^ × 10^−6^ of the patient’s first morning urine in a special sterile plastic container. Plastic tubes with the samples of blood (2 m^3^ × 10^−6^) and urine (5 m^3^ × 10^−6^) were stored at +5 °C until analysis.

### 2.4. Arsenic, Chromium, and Selenium Concentration Measurements

Prior to analysis, blood and urine samples were diluted 1:10 using 5 kg ×10^−3^/m^3^ × 10^−3^ NH_4_OH (Fluka, Sigma Aldrich, St. Louis, MO, USA) + 0.5 kg×10^−3^/m^3^×10^−3^ Triton X-100 (Merck, Darmstadt, Germany) + 0.5 kg × 10^−3^/m^3^ × 10^−3^ EDTA (Fluka, Sigma Aldrich, St. Louis, MO, USA) for blood and 1% nitric acid for urine. To determine urinary arsenic, blood chromium, and selenium concentration, an inductively coupled plasma mass spectrometer NexION 300D ICP-MS (PerkinElmer, Shelton, CT, USA) was used. A simultaneous analysis of a standard reference urine (ClinChek^®^ Urine Control Level 2, Recipe, Munich, Germany) was carried out to ensure the accuracy and precision of the analytical procedures for arsenic concentration in urine. In the reference urine, the certified control range value for arsenic was 66.6–99.9 kg × 10^−9^/m^3^ × 10^−3^. Analyzing the reference urine (*n* = 12), we obtained mean and standard deviation (SD), and the coefficient of variation values of 87.30 ± 6.22 kg × 10^−9^/m^3^ × 10^−3^ and 7.1%, respectively. For blood analysis we used ClinChek^®^ Whole Blood Control Level 3 (Recipe, Munich, Germany). In the reference whole blood, the certified control range values for chromium and selenium were 8.74–13.10 kg × 10^−9^/m^3^ × 10^−3^ and 13.5–20.3 kg × 10^−9^/m^3^ × 10^−4^. Analyzing the reference whole blood (*n* = 11), we obtained mean and SD, and the coefficient of variation values of 11.43 ± 1.55 kg × 10^−9^/m^3^ × 10^−3^ and 13.6% for chromium, and 14.13 ± 1.44 kg × 10^−9^/m^3^ × 10^−4^ and 10.2% for selenium, respectively. We used low-sorption plastic tubes and labware prepared according to previously described protocol [38].

### 2.5. Measurements of Creatinine

To control kidney function [39], we measured creatinine in urine by the Jaffe method [40]. To assess possibly different effects of kidney on arsenic level in urine, we expressed urinary arsenic levels as urine arsenic/urine creatinine (kg × 10^−^^9^/kg × 10^−3^ creatinine).

### 2.6. Statistical Analysis

We summarized baseline characteristics of AD cases and controls computing median and 25 and 75 percentiles for continuous variables with non-normal distributions (age, creatinine-adjusted urinary arsenic level, blood chromium, blood selenium, urinary creatinine), and frequencies for categorical variables. To compare age, creatinine-adjusted urine arsenic, blood chromium, blood selenium, and urinary creatinine between cases and controls, we used the Wilcoxon rank-sum test. Chi-squared test for categorical variables was used to compare characteristics between cases and controls.

To assess interrelationships between metals and metalloids in AD patients, we made Spearman correlation analysis.

We used unconditional logistic regression to estimate the association between urinary arsenic, blood chromium, blood selenium, arsenic–selenium, or chromium–selenium and AD, calculating odds ratios (ORs) and 95% confidence intervals (CI). We adjusted the first model for possible risk factors of AD such as age and gender (female, male), and used further adjustment for education (primary, secondary or specialized secondary, some university or higher), hypertension (absent, present), and cardiovascular diseases and stroke (absent, present) in the second model. The metals arsenic, chromium, and selenium in biological media were categorized using terciles or the medians of their distribution in the controls.

The level of statistical significance was set at 0.05. All reported *p*-values are two-sided. To perform analyses, we used STATA/IC 15.

## 3. Results

AD patients (cases) and controls were different according to age, gender, and education (Table 1). Chronic diseases such as arterial hypertension, cardiovascular diseases and stroke, kidney diseases, depression, and head trauma were more prevalent in AD patients. Prevalence of diabetes mellitus and thyroid diseases did not differ between cases and controls. Cases were less likely to smoke, drink alcohol, and exercise regularly.

In AD patients, urinary arsenic and blood chromium levels were significantly higher, but blood selenium was significantly lower than that in controls (Table 1).

Spearman correlation showed a significant negative relationship between urinary arsenic and blood selenium (r = −0.274, *p* = 0.047) and a suggestive negative relationship between blood chromium and selenium (r = −0.255, *p* = 0.065).

The association of urinary arsenic with the risk of AD was not significant after adjustment for age and other risk factors (Table 2). A significant increase in the odds of AD associated with increased blood chromium was seen in both adjusted models. After final adjustment, blood chromium per 1 kg × 10^−9^/m^3^ × 10^−3^ increment was associated with an OR of 2.39 (95% CI 1.32–4.31). Increased blood selenium was related to a significant decrease in the odds of AD after adjustment for risk factors. Blood selenium per 1 kg × 10^−9^/m^3^ × 10^−4^ increment was associated with 1.4 times lower risk of AD (OR = 0.71; 95% CI 0.58–0.87) (Table 2).

From multivariable logistic regression, the AD association with low blood selenium and high urinary arsenic was not significant (OR = 3.02; 95% CI 0.67–13.72) (Table 3). However, low selenium and high chromium content in blood was associated with a suggestive increase in the odds of AD compared to those with high selenium and low chromium (OR = 3.89; 95% CI 0.98–15.53).

## 4. Discussion

In this study, AD patients had greater urinary arsenic content compared to controls. However, the findings did not show a significant AD association with urinary arsenic after adjustment for possible risk factors. Similar findings on the AD association with total arsenic in urine was reported by Yang et al. [41]. However, further analysis of different arsenic profiles in urine showed inorganic arsenic and arsenic metabolite monomethylarsonous acid being associated with increased risk of AD. The findings on arsenic relation to cognitive function are not consistent [42,43]. The studies that analyzed serum arsenic did not find an association with AD [44,45], while greater arsenic in nail and hair of AD patients was reported [46]. There is evidence that arsenic accumulates in the brains of rodents [47,48] and humans [49,50]. Exposure to arsenic causes brain damages such as neuronal necrosis and apoptosis [51], oxidative stress [52], Tau hyperphosphorylation [28], and transcriptional activation of Aβ precursor protein gene [53]. The main pathways contributing to arsenic-induced neurotoxicity are oxidative stress, increased apoptosis in cerebral neurons, and increased axonal degeneration [54].

We identified a positive association between blood chromium concentration and AD: in the adjusted model; the odds ratio per 1 kg × 10^−9^/m^3^ × 10^−3^ chromium increment was 2.39. After adjustment for age, gender, and other risk factors, a significant association of AD with chromium was reported by Baum et al. [44]. The findings on chromium concentration in AD patients are not consistent; one study presented increased chromium concentrations in AD patients compared to controls [44], the other decreased [55]. Some studies have not reported a difference in concentration [56,57]. Experimental studies show that chromium entering via the digestive system is absorbed more by the brains, liver, and kidneys [58], while entering via respiratory system results in deposits in the lungs [59]. As chromium, similar to other redox active metals, has the ability to produce reactive radicals, disruption of metal ion homeostasis may lead to oxidative stress and reactive oxygen species-related DNA damage, lipid peroxidation, and protein modification, i.e., effects typical for AD and other chronic noncommunicable diseases [31].

This study showed a significant inverse association between blood selenium concentration and AD: blood selenium per 1 kg × 10^−9^/m^3^ × 10^−4^ increment was associated with 1.4 times lower risk of AD. In AD patients, a suggestive inverse selenium–chromium and selenium–arsenic interrelation was supported by a suggestive increase in a risk of AD in individuals with low selenium and high chromium level after adjustment for other risk factors. Reduced concentration of selenium as an antioxidant could be associated with AD because of oxidative stress induced by other metals/metalloids (chromium, arsenic, etc.). Experimental studies show a decisive role of selenium in AD pathogenesis via abilities to reduce mitochondrial oxidative stress [60], to inhibit metal-induced Aβ aggregation [33] and to reduce Tau hyperphosphorylation [61,62]. Meanwhile, the findings of epidemiological studies are inconsistent. A case–control study did not find an association of AD risk with blood selenium. However, individuals with low selenium and high inorganic arsenic had significantly higher risk of AD compared to those with high selenium and low inorganic arsenic level [41]. One recently published cross-sectional study on blood selenium in the elderly demonstrated a reduced risk of mild cognitive impairment for individuals with higher blood selenium [63], but the other did not [42]. Randomized clinical trial transformed into a cohort study did not demonstrate an association of selenium supplement use and AD [64].

Among the studies that determined selenium concentrations in AD patients/controls, some reported lower plasma/serum/blood selenium in AD patients compared to controls [55,57,65,66,67,68,69,70], some did not find a difference [71,72,73,74,75], and one study found higher selenium in AD patients [76]. However, meta-analysis of serum/blood selenium in AD patients defined a decrease in the selenium level correlated with glutathione peroxidase in AD patients [77].

The inconsistency of the findings presented, especially in epidemiological studies, can be explained by several reasons. One of them is the limited number of people involved in the various studies, which may prevent finding a potential difference or reliable results. Another reason could be related to the multi-causality of AD and inclusion or not of risk factors of the disease in the statistical analysis. Interactions between different factors, especially environmental and lifestyle, and different methods used can also affect results.

We used unconditional logistic regression (adjusted for age, gender, education, arterial hypertension, cardiovascular diseases, and stroke) to estimate associations. Because of the limited number of cases, a limited number of variables, that were most prevalent among AD patients, were included in the analysis. The choice of variables was also reinforced by the opportunity to verify information in medical history.

One of the strengths of this study is that exposure to arsenic, chromium, and selenium was assessed by metal/metalloid content in urine and blood. By measuring metals/metalloids in the same laboratory under the same conditions and using the same quality standards for both cases and controls, we avoided one of the greatest limitations of case–control studies related to recall bias in exposure assessment.

In this study, we measured total arsenic in urine to assess recent exposure to the metal [78]. However, we were not able to perform speciation analyses of arsenic due to technical capabilities. Therefore, urinary inorganic arsenic, which is a major toxicant, and its metabolites (monomethylarsonic acid (MMA) and dimethylarsenic (DMA)) used for risk assessment [79,80] were not determined. Despite this, the most common biomarker of exposure for inorganic arsenic is total urinary arsenic, which can be confounded by consuming seafood [80]. Due to the inability to verify the information on diet and dietary habits, the consumption of seafood was not included in regression models. However, we do not think that it could influence the association of AD with urinary arsenic because (1) arsenic in the patient’s urine did not exceed the normal human level (<100 kg × 10^−9^/m^3^ × 10^−3^) [81], when it increases to more than 1000 kg × 10^−9^/m^3^ × 10^−3^ after seafood consumption [78,79]; (2) the use of fish and seafood is very low in Lithuania: in 2019, about half (46.2%) of adults consumed fish 1–2 times per month [82]; meanwhile, the consumption of seafood is even lower, especially among the elderly [83]. Despite this, both total arsenic and arsenic species (inorganic arsenic, its metabolites MMA and DMA, and arsenobetaine) in urine represents a recent exposure [78], while AD is a chronic disease. Therefore, for further studies, questions remain as to how urinary arsenic (total or speciated) can be used to assess long-term exposure to inorganic arsenic [80], and to what extent arsenic contributes to AD ethiopathogenesis.

In this study, chromium and selenium in blood were determined to assess individual exposure to the metals. Chromium can be divalent, trivalent, or hexavalent. Due to the oxidation or reduction reaction, hexavalent chromium can be reduced to trivalent chromium, which is an essential element, and small amounts of trivalent chromium can be oxidized to hexavalent, which is a human carcinogen [79]. However, because of trivalent chromium’s very low ability to enter the cell, the measurement of intracellular chromium is supposed to be a reasonably reliable measure of exposure to hexavalent chromium [59,84].

A potential limitation of the study is the relatively small number of patients. Therefore, some of our estimates of associations have wide confidence intervals, limiting the possibility to draw firm conclusions.

Another limitation is related to recall bias common to case–control studies, particularly in the studies of AD risk factors. Therefore, in logistic regression models, we used only variables verified in medical history.

## 5. Conclusions

Despite some limitations, the study provides evidence that selenium reduces the risk of Alzheimer’s disease whereas chromium increases it. However, further experimental and epidemiological studies, especially cohort studies that can estimate exposure to different risk factors of the disease, would be very useful in the assessment of Alzheimer ’s disease causality.

## Figures and Tables

**Table 1:** Characteristics of Alzheimer’ disease cases and controls.

Variable	Cases (*n* = 53)	Controls (*n* = 217)	*p*-Value for Difference
**Age (years) (median, Q1, Q3)**	85.70 (80.84–88.38)	71.19 (61.13–79.24)	<0.001
**Gender (*n*, %)**			0.002
Male	12 (22.6)	100 (46.1)	
Female	41 (77.4)	117 (53.9)	
**Education (*n*, %)**			<0.001
Primary education	21 (41.2)	50 (23.1)	
Secondary (specialized secondary)	12 (23.5)	127 (58.8)	
Some university or higher	18 (35.3)	39 (18.1)	
**Arterial hypertension (*n*, %)**	45 (84.9)	96 (44.2)	<0.001
**Cardiovascular diseases & stroke (*n*, %)**	40 (75.5)	31 (14.3)	<0.001
**Chronic kidney disease (*n*, %)**	14 (26.4)	15 (6.9)	<0.001
**Thyroid diseases (*n*, %)**	14 (26.4)	34 (15.7)	0.067
**Diabetes mellitus (*n*, %)**	6 (11.3)	22 (10.1)	0.800
**Depression (*n*, %)**	12 (22.6)	6 (2.8)	<0.001
**Head trauma (*n*, %)**	14 (26.4)	14 (6.5)	<0.001
**Ever alcohol users (*n*, %)**	38 (74.5)	200 (92.6)	<0.001
**Ever smokers (*n*, %)**	7 (13,7)	82 (38.0)	0.001
**Exercised regularly**	8 (16.0)	101 (47.0)	<0.001
**Blood chromium (kg × 10^−9^/m^3^ × 10^−3^) (median, Q1, Q3)**	0.7 (0.45–0.99)	0.54 (0.41–0.67)	0.005
**Blood selenium (kg × 10^−9^/m^3^ × 10^−4^) (median, Q1, Q3)**	10.97 (8.74–12.46)	13.53 (11.45–16.19)	0.001
**Urinary arsenic / urinary creatinine (kg × 10^−9^/kg × 10^−3^) (median, Q1, Q3)**	43.07 (24.66–63.43)	32.61 (20.26–46.85)	0.023
**Urinary creatinine (kg × 10^–6^/m^3^ × 10^−4^) (median, Q1, Q3)**	81.15 (50.04–110.91)	101.28 (65.54–159.72)	0.003

Abbreviations: Q_1_, 25 percentile; Q_3,_ 75 percentile.

**Table 2:** Odds ratios (ORs) and 95% confidence intervals (CIs) for the association of creatinine-adjusted urinary arsenic (kg × 10^−9^/kg × 10^−3^ creatinine), blood chromium (kg × 10^−9^/m^3^ × 10^−3^), and blood selenium (kg × 10^−9^/m^3^ × 10^−4^) with Alzheimer disease.

Variables	Cases	Controls	OR (95% CI) ^a^	OR (95% CI) ^b^
	*n* (%)	*n* (%)		
**Urinary arsenic**				
≤23.59	13 (24.6)	71 (32.7)	1.0	1.0
23.60–41.18	12 (22.6)	72 (33.2)	0.80 (0.29–2.17)	0.96 (0.28–3.26)
≥41.19	28 52.8)	74 (34.1)	1.29 (0.53–3.15)	2.01 (0.66–6.07)
Continuous (per 1 kg × 10^−9^/kg × 10^−3^ creatinine)	53 (100.0)	217 (100.0)	1.00 (0.99–1.01)	1.00 (0.99–1.02)
**Blood chromium**				
≤0.45	13 (24.5)	64 (32.8)	1.0	1.0
0.46–0.61	12 (22.6)	64 (32.8)	0.83 (0.29–2.34)	1.23 (0.33–4.58)
≥0.62	28 (52.8)	67 (34.4)	2.47 (0.99–6.15)	4.46 (1.39–14.30)
Continuous (per 1 kg × 10^−9^/m^3^ × 10^−3^)	53 (100.0)	195 (100.0)	1.93 (1.19–3.13)	2.39 (1.32–4.31)
**Blood selenium**				
≤13.53	46 (86.8)	99 (50.8)	1.0	1.0
≥13.54	7 (13.2)	96 (49.2)	0.31 (0.12–0.79)	0.32 (0.11–0.95)
Continuous (per 1 kg × 10^−9^/m^3^ × 10^−4^)	53 (100.0)	195 (100.0)	0.75 (0.64–0.87)	0.71 (0.58–0.87)

^a^ Adjusted for age and gender. ^b^ Further adjustment for education, arterial hypertension, cardiovascular diseases, and stroke.

**Table 3:** Odds ratios (ORs) and 95% confidence intervals (CIs) for the association between blood selenium (kg × 10^−9^/m^3^ × 10^−4^) combination either with blood chromium (kg × 10^−9^/m^3^ × 10^−3^) or creatinine-adjusted urinary arsenic (kg × 10^−9^/kg × 10^−3^ creatinine) and Alzheimer disease *.

Variables	Cases	Controls	OR (95% CI) ^a^	OR (95% CI) ^b^
	*n* (%)	*n* (%)		
**Blood Se ^1^ & urinary As** ^2^				
High Se & low As	4 (7.6)	44 (22.6)	1.0	1.0
High Se & high As	3 (5.7)	52 (26.7)	0.57 (0.11–2.94)	0.36 (0.05–2.72)
Low Se & low As	14 (26.4)	55 (28.2)	1.67 (0.44–6.33)	1.00 (0.20–5.13)
Low Se & high As	32 (60.4)	44 (22.6)	3.18 (0.90–11.22)	3.02 (0.67–13.72)
**Blood Se & blood Cr** ^3^				
High Se & low Cr	5 (9.4)	51 (26.2)	1.0	1.0
High Se & high Cr	2 (3.8)	45 (23.1)	0.52 (0.09–3.08)	0.38 (0.05–3.16)
Low Se & low Cr	16 (30.2)	50 (25.6)	1.76 (0.52–5.97)	1.09 (0.25–4.68)
Low Se & high Cr	30 (56.6)	49 (25.1)	3.47 (1.07–11.20)	3.89 (0.98–15.53)

* The metals—selenium (Se), arsenic (As), and chromium (Cr)—in blood or urine were categorized using the medians of their distribution in the controls. ^1^ Low Se: ≤13.5 kg × 10^−9^/m^3^ × 10^−4^; high Se: ≥13.54 kg × 10^−9^/m^3^ × 10^−4^. ^2^ Low As: ≤32.61 kg × 10^−9^/m^3^ × 10^−3^; high As: ≥32.62 kg × 10^−9^/m^3^ × 10^−3^. ^3^ Low Cr: ≤0.54 kg × 10^−9^/m^3^ × 10^−3^; high Cr: ≥0.55 kg × 10^−9^/m^3^ × 10^−3^. ^a^ Adjusted for age and gender. ^b^ Further adjustment for education, arterial hypertension, cardiovascular diseases, and stroke.

## Data Availability

The data presented in this study are available on reasonable request from the corresponding author. The data are not publicly available due to the absence of such possibility in ethical approval.
